# Behavioural responses of *Frankliniella occidentalis* Pergande larvae to methyl jasmonate and *cis*-jasmone

**DOI:** 10.1007/s10340-013-0532-8

**Published:** 2013-10-15

**Authors:** Barbara Egger, Elisabeth H. Koschier

**Affiliations:** Division of Plant Protection, Department of Crop Sciences, University of Natural Resources and Life Sciences (BOKU), Vienna, Austria

**Keywords:** Thrips, Second instar, Secondary plant compounds, Jasmonates, Feeding deterrent, Leaving behaviour

## Abstract

The larval stages of *Frankliniella occidentalis* Pergande (Thysanoptera: Thripidae) cause more direct feeding damage to plants than the adults. We, therefore, investigated the behaviour-modifying effects on second instar larvae of two jasmonic acid derivatives. The artificial application of methyl jasmonate and *cis*-jasmone, both at 1 % concentration, deterred the larvae from settling in a dual choice bean leaf disc assay. We observed a dose-dependent feeding deterrence of both jasmonates and calculated the concentration required to reduce the feeding damage by 50 % relative to the control treatment (FDC_50_) for each jasmonate. The feeding damage was reduced by the application of *cis*-jasmone at 1 % concentration, but not by the jasmonates at the respective FDC_50_ in no-choice leaf disc bioassays. However, significantly more larvae left jasmonate-treated whole potted bean plants by migrating to the soil compared with control plants. Our results may be exploited extending behavioural manipulation by using plant compounds in thrips control programmes to the full lifecycle of the pest. Plant compounds could be used in integrated and biological pest management strategies against *F. occidentalis* in combination with the application of various above and below ground control measures.

## Introduction

*Frankliniella occidentalis* Pergande (Thysanoptera: Thripidae), the western flower thrips, is a major pest in agricultural and horticultural crops worldwide (Kirk and Terry 2003). The adults and larvae of this highly polyphagous pest insect feed on epidermal and sub-epidermal plant tissues, causing necrotic blotches and reducing their photosynthetic capacity. In addition, the plants are damaged indirectly by the transmission of various plant viruses (Moritz 2006). *Frankliniella occidentalis* exhibits a thigmotactic behaviour that makes an efficient application of chemical insecticides difficult. Furthermore, some thrips pests have developed resistance to a range of insecticides (Jensen 2000; Reitz 2009). Several beneficial organisms whose target instars are predominantly the larval instars of the thrips, e.g. predatory mites, parasitic wasps or entomopathogenic fungi, are successfully used in biological control measures or are currently investigated for thrips control (Brodsgaard 2004; Ebssa et al. 2006).

Methyl jasmonate and *cis*-jasmone are constituents of essential oils, for instance, from *Jasminum* sp., *Lonicera* sp. or *Philadelphus* sp. (Mookherjee et al. 1990; Joulain 1986). Moreover, being stress-related secondary plant compounds, both jasmonic acid derivatives are known to play a major role in plant defence mechanisms against herbivores (Birkett et al. 2000; Howe and Jander 2008). Exogenous applications of *cis*-jasmone or methyl jasmonate to plants in laboratory and field studies have been shown to induce plant resistance to herbivores and indirectly affected pests such as various aphid species (e.g. Bruce et al. 2003a, b; Brunissen et al. 2010; Glinwood et al. 2007; Thaler et al. 2001), the two-spotted spider mite *Tetranychus urticae* Koch (Rohwer and Erwin 2010) and *F.* *occidentalis* adults (Thaler et al. 2001). Furthermore, methyl jasmonate and *cis*-jasmone may act as an indirect defence mechanism of plants by attracting natural enemies of the herbivores, among them being the aphid parasitoid *Aphidius ervi* Haliday and other hymenopteran parasitoids (Bruce et al. 2003b; Simpson et al. 2011).

Research on the direct effects of jasmonates on herbivores is comparatively scarce. Olfactometer studies revealed repellence of *cis*-jasmone to the grain aphid *Sitobion avenae* Fabricius (Bruce et al. 2003a, b) and the lettuce aphid *Nasonovia ribis*-*nigri* Mosley. Traps baited with *cis*-jasmone were found repellent to the damson-hop aphid *Phorodon humuli* Schrank (Birkett et al. 2000), but attracted the pest thrips species New Zealand flower thrips (*Thrips obscuratus* Crawford) (Teulon et al. 2007) in field experiments.

In contrast, some previous findings indicate that western flower thrips adults respond negatively to jasmonates: *F.* *occidentalis* females avoided methyl jasmonate-treated chrysanthemum plants in the laboratory (Bruhin 2009), significantly lower numbers of thrips were found on jasmonic acid-sprayed plants in the field (Thaler et al. 2001) and jasmonate-baited traps did not attract *F.* *occidentalis* (James 2005). These studies in particular and most other studies on the effects of secondary plant volatiles on thrips, in general, have mainly focused on adults. Possible direct effects on larvae have yet to be researched. This topic is of considerable interest, because *F.* *occidentalis* larval stages usually cause more direct feeding damage than adults because of their greater abundance on a plant (Wiesenborn and Morse 1986). However, if and how the less-mobile larvae of *F.* *occidentalis* respond to the jasmonates is not known to date.

The aim of our study was therefore to investigate the potential deterrent effects of an application of *cis*-jasmone and methyl jasmonate to bean leaf discs and potted bean plants on *F.* *occidentalis* second instar larvae. Initially, we observed the settling preference of the larvae for treated or untreated leaf discs in choice bioassays. Second, we determined the concentration required to reduce the feeding damage by 50 % relative to the control treatment (FDC_50_) for either test dilution and evaluated their feeding deterrent activity to the larvae in a no-choice leaf disc assay. Finally, we investigated the larval behaviour on whole bean plants sprayed with the test dilutions.

## Materials and methods

### Insects and plants

*Frankliniella* *occidentalis* were reared on detached bean leaves on 1 % water agar (Agar, Sigma-Aldrich, Vienna, Austria) in plastic Petri dishes (14-cm diameter) in a climate chamber at 24 ± 1 °C, 45 ± 5 % relative humidity and a L16:D8 photoperiod. The dishes were closed with lids with central holes covered with a fine mesh to ensure ventilation. To obtain groups of second instar larvae of known age for the bioassays, *F.* *occidentalis* females were allowed to lay eggs on bean leaves in separate Petri dishes for 24 h. The larvae were used for testing 5–6 days after the end of the oviposition.

Bean plants (*Phaseolus vulgaris*, var. Borlotto) used for rearing as well as for testing were grown in a plant growing room at 25 ± 1 °C, at 50 ± 5 % relative humidity and at a L16:D8 photoperiod in groups of 13–15 plants per pot. Leaf discs used for the bioassays were punched from fully developed leaves of 11–13 days old bean plants by means of a cork borer. The potted bean plants were grown singly and were used for the experiment after having fully developed both primary leaves.

### Application of compounds

The test compounds methyl jasmonate (≥95 %, Sigma-Aldrich, Vienna, Austria) and *cis*-jasmone (≥85 %, Sigma-Aldrich, Vienna, Austria) were diluted in pure ethanol (Merck, Darmstadt, Germany) at a ratio of 1:10 and in a relative quantity of distilled water mixed with a surfactant (0.05 % Triton X-100, Sigma-Aldrich, Vienna, Austria) to obtain a range of concentrations (0.1–1 %). The control solution consisted of ethanol (Merck, Darmstadt, Germany) and distilled water with the surfactant (0.05 % Triton X-100, Sigma-Aldrich, Vienna, Austria) at a ratio of 1:10.

For the leaf disc bioassays, the bean leaf discs were sprayed with the respective solution using a Potter Precision Laboratory Spray Tower (Burkard Manufacturing Co Ltd., Rickmansworth, UK) resulting in a solution quantity on the leaf of about 1 μl/cm². The treated leaf discs were allowed to dry for approximately 10 min and subsequently placed on 1 % water agar (Fluka Analytical, Sigma-Aldrich, Vienna, Austria) droplets in a Petri dish to prevent the leaf discs from wilting. After releasing the test insects, the bioassay units were closed with a plastic sealing film (Carl Roth, Karlsruhe, Germany) which was perforated subsequently with insect pins to ensure ventilation (approximately 10 holes per cm²).

The upper and lower leaf surfaces of the whole bean plants used for the larval leaving bioassays were sprayed with a total amount of 2.5 ml of methyl jasmonate, *cis*-jasmone (either at the FDC_50_, see below) or control solution per plant by means of an airbrush (DeVilbiss, Bournemouth, UK) resulting in a similar solution quantity to the one for the leaf disc assay. The solution quantity was determined by comparing the spray distributions of the potter spray tower and the airbrush using water-sensitive paper (Syngenta, Basel, Switzerland). The solutions were allowed to dry for approximately 10 min before releasing the test insects.

### Settlement preference

Choice experiments were conducted to test for settlement preferences of *F.* *occidentalis* larvae at 1 % compound concentration. A control bean leaf disc and a treated leaf disc (1.6-cm diameter each) were placed at a distance of approximately 4 cm to each other on two 350 μl water agar droplets in a glass Petri dish (6-cm diameter) to prevent the leaf discs from wilting. A group of 10 *F.* *occidentalis* second instar larvae was released in the centre of the Petri dish. The dish was sealed with a perforated plastic sealing film as described above. After 30 min, 1, 2, 3, 4, 5 and 6 h the positions of the larvae on either leaf disc or elsewhere in the Petri dish were recorded. Each treatment was replicated with 12–14 groups of larvae.

### Feeding deterrence index

In a choice bioassay, a control bean leaf disc and a treated leaf disc (1.1-cm diameter each) were placed on two 350 μl water agar droplets in a glass Petri dish (6-cm diameter) at a distance of approximately 4 cm to each other to prevent the leaf discs from wilting. A single second instar larva was released in the centre of the Petri dish. The dish was sealed with a perforated plastic sealing film as described above. After 24 h, the larva was removed, and the feeding damage was measured using a transparent counting grid (0.25 × 0.25 mm) and a stereo microscope. This procedure was repeated with four different concentrations (0.1, 0.25, 0.5 and 1 %) of each test compound for calculating a feeding deterrence index (FDI) using the formula:

$${\text{FDI}} = 100[(C - T)/(C + T)]$$

where *C* and *T* are the control and treated leaf areas damaged by the larvae (Isman et al. 1990). The bioassay was replicated 25–35 times per concentration for each compound.

### Assessment of feeding damage

For this bioassay, a single leaf disc (1.6-cm diameter) treated either with methyl jasmonate, *cis*-jasmone (both at the respective FDC_50_ or at 1 % concentration) or control solution was placed on a 700 μl water agar droplet in a glass Petri dish (6-cm diameter) to prevent the leaf discs from wilting. A group of 10 *F.* *occidentalis* second instar larvae was released on the leaf disc, and the dish was sealed with a perforated plastic sealing film as described above. After 24 h, the larvae were removed, the feeding damage was measured using a transparent counting grid (0.25 × 0.25 mm) and a stereo microscope and subsequently converted into the percentage of damaged area on the leaf discs. The test was replicated with 13–14 groups of larvae per treatment.

### Larval leaving behaviour

A bioassay adapted from Teerling et al. (1993) was conducted to test the hypothesis that *F.* *occidentalis* larvae would reject a jasmonate treated plant by leaving it. Black cardboards (20 × 20 cm) coated with a sprayable insect trapping adhesive (Souverode, Witasek Pflanzenschutz GmbH, Austria) covered the pots for entrapping larvae that drop from the plant. In addition, insect glue (Raupenleim, Stähler Austria GmbH & Co KG, Austria) was applied around the stem 2 cm above and below the leaf nodes for trapping the larvae that leave the plants by walking down the stem. Groups of 30 *F.* *occidentalis* second instar larvae per plant were released on the leaves using a fine brush. After keeping the differently treated plants for 24 h in separate climate chambers at 24 ± 1 °C, 45 ± 5 % relative humidity and a L16:D8 photoperiod, larvae on the adhesive and on the plant were counted. The bioassay was replicated with eight plants per treatment.

### Statistical analysis

*T* tests were performed to compare the numbers of settled larvae and the area of feeding damage on the leaf discs. The FDI was calculated using the mean feeding damage at four different concentrations of each compound. FDC_50_ (the concentration that reduced feeding damage on the treated leaf disc by 50 % compared with the control leaf disc) was calculated via linear regression of percentage feeding deterrence after log-transforming the concentrations for the line of best fit (Seffrin et al. 2010). Data on larvae leaving treated or untreated plants were analysed using a one-way ANOVA and a Bonferroni post hoc test. All statistical analyses were performed using the statistical software PASW 18.0.0 (SPSS Inc., USA).

## Results

### Settlement preference

**Fig. 1:**
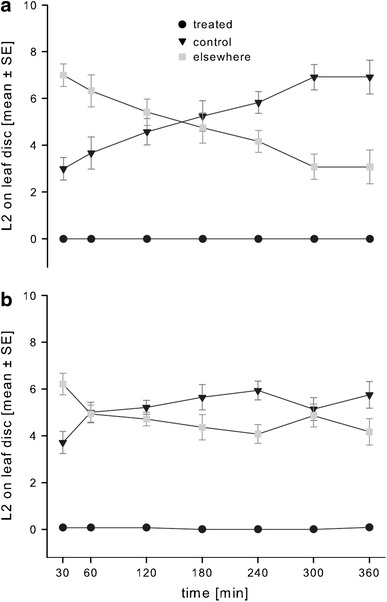
Mean number (±SE) of settled *F.* *occidentalis* second instar larvae on either control leaf discs, treated leaf disc or elsewhere in the bioassay unit. Dual choice bioassay with leaf discs treated with **a** methyl jasmonate or **b** *cis*-jasmone (both at 1 %) and a control leaf disc. Settlement preference recorded at 30 min, 1, 2, 3, 4, 5 and 6 h after the release of groups of 10 *F.* *occidentalis* second instar larvae per unit, 12–14 groups tested

The number of *F.* *occidentalis* larvae settling on one of the two leaf discs in the bioassay unit gradually increased: After 30 min, more than 30 % of the larvae had chosen one of the leaf discs in the methyl jasmonate bioassay unit, whereas after 6 h, about 70 % of the larvae had settled on one of the leaf discs (Fig. 1a). Similarly, 37 % of the larvae had chosen one of the leaf discs in the *cis*-jasmone bioassay unit after 30 min, whereas after 6 h, about 60 % of the larvae had settled on one of the leaf discs (Fig. 1b).

Either jasmonate applied at 1 % concentration exhibited a highly significant deterrent effect on the *F.* *occidentalis* larvae (Fig. 1). At the most one larva per bioassay unit was counted on the leaf disc treated with *cis*-jasmone (paired *t* test, *P* < 0.001). Methyl jasmonate-treated leaf discs were avoided by the larvae at any of the observed points in time (paired *t* test, *P* < 0.001).

### Feeding deterrence

**Fig. 2:**
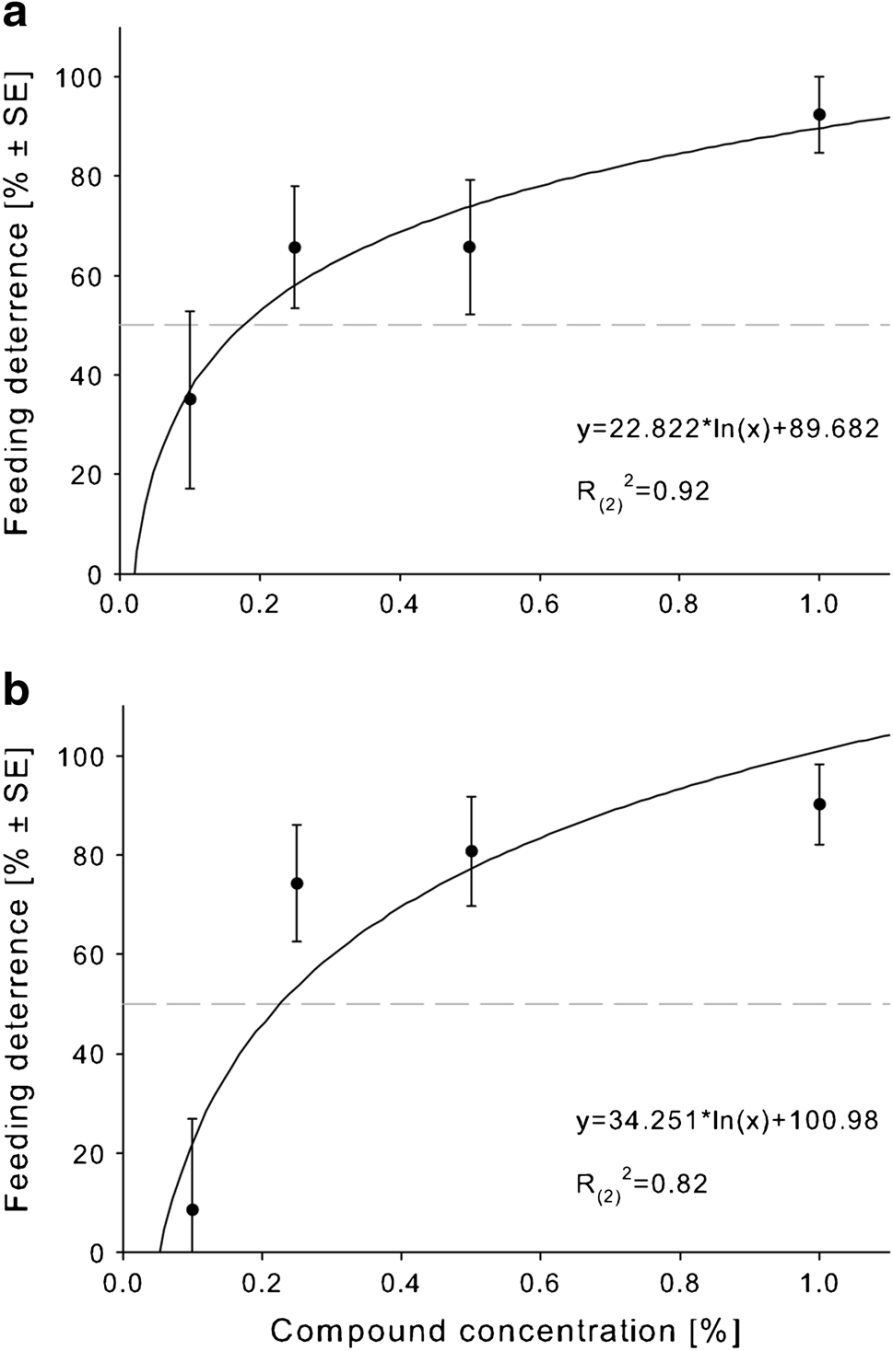
Mean feeding deterrence of **a** methyl jasmonate and **b** *cis*-jasmone to *F.* *occidentalis* second instar larvae at increasing concentrations in a choice bioassay

The feeding deterrent effect of methyl jasmonate, as well as those of *cis*-jasmone on *F.* *occidentalis* larvae, was positively correlated with increasing compound concentrations (Fig. 2). The FDC_50_, i.e. the concentration that reduced the feeding damage by 50 % on a treated leaf disc, was determined at about 0.18 % for methyl jasmonate and at about 0.23 % for *cis*-jasmone.

**Fig. 3:**
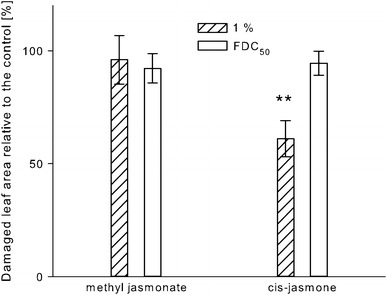
Percentage of damaged area (±SE) caused by the feeding activity of *F.* *occidentalis* second instar larvae on leaf discs treated with methyl jasmonate or *cis*-jasmone (both at 1 % or FDC_50_) relative to the damaged area of control leaf discs in a no-choice test within 24 h. Means are significantly different at **P < 0.01; unpaired *t* test

Leaf discs treated with 1 % *cis*-jasmone reduced feeding damage caused by the larvae in the no-choice situation (unpaired *t* test, *T*_(30)_ = 3.685, *P* < 0.01); however, neither the application of 1 % methyl jasmonate nor of either test dilution at the respective FDC_50_ reduced the feeding damage on treated leaf discs compared with the untreated leaf discs (Fig. 3).

### Larval leaving behaviour

The treatment of bean plants with methyl jasmonate or *cis*-jasmone at 0.18 or 0.23 % concentration (FDC_50_), respectively caused the *F.* *occidentalis* larvae to leave the plants. After 24 h, significantly more larvae were found on the sticky cardboards underneath the jasmonate-treated plants compared to the control plants (one-way ANOVA, F_(2, 21)_ = 20.305, *P* < 0.001). While about 30 % of the larvae left the methyl jasmonate or *cis*-jasmone-treated plants, only about 11 % left the control plants (Table 1).

**Table 1:** Mean number (±SE) per plant of *F.* *occidentalis* second instar larvae leaving bean plants treated with the concentration required to reduce the feeding damage by 50 % (FDC_50_) of methyl jasmonate, respectively *cis*-jasmone and the control solution within 24 h. 30 larvae were released per plant

Treatment	Number of larvae leaving the plant		
	Mean	±SE	
Control	3.25	±0.49	A
Methyl jasmonate 0.18 %	9.25	±0.63	B
*Cis*-jasmone 0.23 %	10.13	±0.73	B

Values followed by different letters are significantly different (one-way ANOVA, Bonferroni)

## Discussion

We focussed our study on *F.* *occidentalis* second instar larvae because, due to their high abundance and feeding commitment, the immature stages are responsible for a large part of the direct feeding damage on plants (Wiesenborn and Morse 1986). As far as we know, only one study with antifeedants was carried out with thrips larvae so far: carvacrol and thymol, two monoterpenoid phenols, applied to the test plants at a concentration of 1 % deterred *F.* *occidentalis* second instar larvae in choice bioassays. In addition, the feeding damage of the larvae was reduced in a no-choice situation (Peneder and Koschier 2011).

In the first step, we used *cis*-jasmone and methyl jasmonate both at 1 % concentration in choice settlement assays to examine these substances for potential deterrent effects since this compound concentration proved to be efficient to screen for other potential deterrent substances in earlier studies (Koschier et al. 2007; Riefler and Koschier 2009). We identified both jasmonates as deterrents to the larvae: At each point of time during the observation, more than 90 % of the larvae avoided contact with the jasmonate-treated leaf discs. This highly significant settlement preference for the control leaf discs suggests that, although *F.* *occidentalis* larvae are not the host seeking instar (Terry 1997), they are able to respond to deterrents applied to leaf discs.

In the second step, we determined a feeding deterrent index to detect the range of effectiveness of the jasmonates. In the present study both jasmonates proved to be dose-dependent deterrents to thrips larvae. The calculation of the FDC_50_, the deterrent concentration required to reduce feeding damage by 50 % relative to the control, allows a direct comparison of the effectiveness of different compounds. However, the feeding damage was not reduced after the application of the FDC_50_ of either jasmonate when the larvae had no choice. Only a relatively high 1 % concentration resulted in a reduction of the feeding damage after the application of *cis*-jasmone. This suggests that the commitment to feeding of second instar larvae exceeds the deterrent effect of the jasmonates at lower concentrations when there is no alternative food source available. On a whole treated plant, the larvae would have the alternative choice of leaving the plant and migrating to the soil.

Experiments with potted bean plants revealed that more than 90 % of *F.* *occidentalis* choose the soil as pupation site as late second instar larvae (Berndt et al. 2004a). Deterrent plant compounds applied to their host plants might cause the larvae to leave the treated plant earlier during their development, thus reducing the feeding damage caused by the larvae. After the application of the synthetic thrips alarm pheromone dodecyl-acetate dropping rates of 5–15 %, depending on the method of application, were reported (Teerling et al. 1993). In our study, we achieved dropping rates of about 30 % after the application of the jasmonates, whereas about 10 % left the control plants, presumably to search for an appropriate pupation site in the soil.

We located the larvae that had left the plants on the sticky cardboard traps, but not on the stems coated with adhesive. Therefore, we assume that the larvae dropped to the cardboards, i.e. the soil, rather than walked down the stem towards the soil.

The increased leaving rates indicate that the jasmonates could be integrated in thrips control strategies, for instance, as synergists in the context of push–pull strategies or biological control strategies. Efficient biological control measures against soil-dwelling thrips stages are the application of entomopathogenic fungi *Beauveria bassiana* Balsamo and *Metarhizium anisopliae* Metsch (Ansari et al. 2008; Ugine et al. 2005), soil-dwelling predatory mites *Hypoaspis* sp. and *Stratiolaelaps miles* Berlese (Berndt et al. 2004a, b), and entomopathogenic nematodes (Ebssa et al. 2004). Since the *F.* *occidentalis* larvae are the main target instar for predators and parasitoids, an additional possible synergistic effect of jasmonate application could be the attraction of various above-ground beneficial insects, among them being some hymenopterous parasitoids of thrips (Birkett et al. 2000; Bruce et al. 2003a; James and Grasswitz 2005; James 2005; Simpson et al. 2011; Thaler 1999). Steiner et al. (2011) revealed that the number of thrips larvae dropping from the plants decreased at a relative humidity of over 81 %. The manipulation of the relative humidity could be combined with the application of deterrents and measures against the soil dwelling thrips instars.

Our study is the first to demonstrate larval plant-leaving behaviour after the artificial application of secondary plant volatiles. Methyl jasmonate and *cis*-jasmone exhibit a deterrent effect to *F.* *occidentalis* second instar larvae and cause the larvae to leave bean plants, presumably by dropping to the soil. These direct and indirect effects may be exploited in integrated and biological pest management strategies by combining them with other control measures. Further investigation on the influence of jasmonates on the thrips metabolism, on behavioural responses of adult *F.* *occidentalis* to volatile jasmonates and the potential of artificial jasmonate treatments to induce plant defence mechanisms against Thysanoptera are necessary to evaluate the future role of jasmonates in thrips control strategies.
